# Dosing adjuvant vitamin C in critically ill patients undergoing continuous renal replacement therapy: We are not there yet!

**DOI:** 10.1186/s13054-018-2297-1

**Published:** 2019-01-09

**Authors:** Patrick M. Honore, David De Bels, Luc Kugener, Sebastien Redant, Rachid Attou, Andrea Gallerani, Herbert D. Spapen

**Affiliations:** 1 ICU Department, Centre Hospitalier Universitaire Brugmann-Brugmann University Hospital, 4, Place Arthur Van Gehucthen, 1020 Brussels, Belgium; 2 Ageing & Pathology Research Group, Vrije Universiteit Brussel, 101, Laarbeeklaan, 1070 Brussels, Belgium

We read with great interest the recent letter to *Critical Care* by Marik and Hooper [1]. Vitamin C (vit C) is increasingly recognized as a crucial compound to alleviate morbidity in critically ill patients. Vit C concentrations, however, are usually far below normal and even close to “scurvy levels” in this population. Vit C also is substantially cleared by continuous renal replacement therapy (CRRT). Significant vit C deficiency was observed in 80% of patients subjected to various types of CRRT despite receiving a daily intravenous (IV) supplement of 500 to 1000 mg [2]. Therefore, high-dose (from 6 to 12 g) vit C substitution during CRRT seems justified [3].

Marik and Hooper argued against such dose increase in patients receiving CRRT. To support their statement, they provided serum vit C dosages in a small number of septic patients who received 6 g vit C IV while undergoing continuous veno-venous hemofiltration (CVVH). Vit C trough and peak levels were largely above normal and comparable to levels obtained in patients not receiving CVVH [1].

We want to warn against oversimplification. Marik and Hooper measured vit C within 30 min after the end of vit C infusion. It would have been more relevant to measure vit C after 24 to 48 h of CVVH treatment. Up to 50% of vit C is cleared in a time-dependent manner during a 4-h session of intermittent hemodialysis or hemodiafiltration [4, 5], which suggests that continuous techniques may exacerbate vit C losses. Vit C also is eliminated by both diffusion (dialysis) and convection (filtration). During hemodiafiltration, diffusion is responsible for two thirds of the vit C loss whereas convection accounts only for one third [5]. CVVH is a sheer convective technique in contrast with other often-used CRRT modes in the critically ill, such as continuous veno-venous hemodialysis (CVVHD) and continuous veno-venous hemodiafiltration (CVVHDF). Marik and Hooper thus report the most modest way of CRRT-induced vit C elimination. It is reasonable to think that more diffusion-based CRRT techniques may yield other results.

We agree with Marik and Hooper that 6 g/day vit C IV is sufficient for patients without acute kidney injury and not requiring CRRT. However, vit C measurements should be performed after prolonged CVVH sessions to ensure that a 6 g daily supplement can keep levels within normal range. More studies are needed in patients receiving CVVHD or CVVHDF to exclude overlooking too great a vit C loss.

## Abbreviations

CRRT: Continuous renal replacement therapy
CVVH: Continuous veno-venous hemofiltration
CVVHD: Continuous veno-venous hemodialysis
CVVHDF: Continuous veno-venous hemodiafiltration
IV: Intravenous
Vit C: Vitamin C
